# Early prediction of anastomotic leakage within 24 h after minimally invasive colorectal cancer surgery using postoperative inflammatory markers: development and temporal validation of a machine learning model

**DOI:** 10.1007/s00464-026-13163-z

**Published:** 2026-07-23

**Authors:** Jose Martin-Arevalo, Andreia Guimaraes, Irina Palomo-Lopez, David Moro-Valdezate, Leticia Perez-Santiago, Stephanie Anne Garcia-Botello, Francisco Castillejos-Ibañez, Fernando Lopez-Mozos, Marina Riera-Cardona, David Casado-Rodrigo, Monica Millan, Vicente Pla-Marti

**Affiliations:** 1 University of Valencia, Valencia, Spain; 2 Departament of General and Digestive Surgery, Clinic University Hospital of Valencia, Valencia, Spain; 3 INCLIVA Biomedical Research Institute, Valencia, Spain; 4 Centro Hospitalar E Universitário de Coimbra, Coimbra, Portugal; 5 Department of Colorectal Surgery, University Clinic Hospital of Valencia, Av. Blasco Ibáñez 17, 46010 Valencia, Spain

**Keywords:** Anastomotic leakage, Machine learning, Colorectal surgery, C-reactive protein, SIRI, Temporal validation

## Abstract

**Purpose:**

Early identification of anastomotic leakage (AL) is critical for safe discharge within enhanced recovery pathways. This study developed and prospectively validated a machine learning (ML) model to predict AL using 24-h postoperative inflammatory biomarkers.

**Methods:**

We analyzed 1,961 patients undergoing elective minimally invasive colorectal resection (2012–2025). Five ML architectures were developed using a 70/15/15 split. The Regularized Logistic Regression (RLB) model was selected and locked with a pre-specified threshold (0.1258). Global variable importance and directionality were assessed via SHAP analysis. Prospective temporal validation was performed on 250 consecutive patients (February 2024 – December 2025).

**Results:**

AL incidence was 9.8% in the development cohort. The RLB model achieved high discrimination (AUCPR 0.859; AUC-ROC 0.819). Postoperative C-reactive protein (CRP) and the Systemic Inflammation Response Index (SIRI) at 24 h were the strongest predictors. During temporal validation, despite a 70% relative reduction in AL incidence (2.8%), the model maintained a robust negative predictive value (NPV) of 97.9% (95% CI 95.1–99.1% and an AUC of 0.73 (95% CI 0.54–0.92). Calibration was near-optimal (slope 0.987, intercept 0.505). Decision curve analysis demonstrated superior net clinical benefit across risk thresholds of 5–20%.

**Conclusions:**

ML-based integration of early inflammatory biomarkers provides a reliable "safety filter" for postoperative surveillance. The high NPV supports objective decision-making for early discharge, even in changing clinical environments with decreasing complication rates.

Colorectal cancer remains one of the most frequently diagnosed malignancies worldwide and a leading cause of cancer-related mortality, accounting for approximately 10% of all new oncological diagnoses [1]. Surgical resection with primary anastomosis continues to represent the cornerstone of curative treatment. Despite major advances in surgical technique, perioperative care, and enhanced recovery after surgery (ERAS) protocols [2], anastomotic leakage (AL) persists as the most feared complication following colorectal resection, with reported incidences ranging from 5 to 15% in contemporary series [3–5]. AL is associated with a substantial increase in postoperative morbidity, mortality, length of hospital stay, healthcare costs, and impaired oncological outcomes, including higher rates of local recurrence and reduced long-term survival [6–9].

AL is a multifactorial complication influenced by patient factors, surgical technique, tissue perfusion, microbiological environment, and host inflammatory response. Numerous preoperative, intraoperative, and postoperative risk factors have been described, and several predictive scores have been proposed [10, 11]. However, most existing models rely on linear statistical assumptions and predominantly static variables, resulting in only modest predictive performance and limited clinical utility. Moreover, significant heterogeneity in AL definitions and diagnostic criteria has historically hindered standardization and comparability across studies, despite international efforts to harmonize definitions and reporting standards [4].

Several studies have demonstrated the feasibility and safety of discharge within 24–48 h following minimally invasive colorectal resection in carefully selected patients, highlighting the potential for reduced healthcare resources utilization and improved patient satisfaction [12–16]. However, this paradigm shift inevitably amplifies concerns regarding the early detection of severe postoperative complications, particularly AL, which often manifests beyond the initial postoperative period. Consequently, reliable tools capable of identifying patients at high risk of AL within the first 24 h after surgery are critically needed to ensure safe early discharge and to guide targeted postoperative surveillance.

To date, most biomarkers and predictive models have achieved their highest diagnostic accuracy between postoperative days 2 and 5, when clinical deterioration is frequently already established [17–19]. Although individual early biomarkers, including C-reactive protein, procalcitonin, leukocyte subpopulations, and composite inflammatory indices, have shown promising diagnostic value, their isolated performance at 24 h assessment remains insufficient for robust clinical decision-making. This underscores the need for integrative approaches capable of capturing complex, non-linear interactions among perioperative clinical variables and dynamic inflammatory responses [20].

Machine learning (ML) techniques offer a powerful framework for modeling such complexity, enabling the integration of high-dimensional data and dynamic biomarkers into predictive algorithms. In this context, the incorporation of early postoperative systemic inflammatory indices represents a particularly attractive strategy to enhance early AL prediction and facilitate individualized postoperative management [19–21].

Therefore, the aim of this study was to develop and temporally validate a machine learning model for early prediction of anastomotic leakage within the first 24 h after minimally invasive colorectal resection.

## Materials and methods

### Study design and population

A retrospective development cohort with a prospectively evaluated temporal validation cohort was assembled, including all patients who underwent surgery with curative intent for histologically confirmed colon or rectal adenocarcinoma at the Coloproctology Unit of Hospital Clínico Universitario de Valencia between January 2012 and December 2025. The study was conducted and reported in accordance with the STROBE [22] and TRIPOD guidelines [23].

### Inclusion and exclusion criteria

Eligible patients were those aged 18 years or older with an American Society of Anesthesiologists (ASA) physical status classification ≤ 4, a histological diagnosis of colorectal adenocarcinoma, and who underwent elective, minimally invasive surgery (laparoscopic or robotic) with curative intent. Patients with rectal cancer who received neoadjuvant therapy based on the assessment by the institutional Medical Oncology Department were also included.

Exclusion criteria were conversion to open surgery, procedures performed using a transanal endoscopic approach, and pregnancy.

### Data collection and study variables

Data were sourced from a prospectively maintained institutional database and augmented by a meticulous review of electronic medical records. The dataset encompassed patient demographics (age and biological sex) and baseline health status as measured by the Charlson Comorbidity Index. Disease-related variables included the primary diagnosis, the surgical procedure performed, and the height of the anastomosis. Perioperative factors assessed included oral bowel preparation, antibiotic prophylaxis, operative time (from incision to closure), and the type of anastomosis. Laboratory analysis was based on preoperative full blood count and standard biochemistry panels obtained within 30 days before surgery, as well as postoperative samples drawn at 24 h following the procedure. From these hematological parameters, a panel of systemic inflammation and nutritional indices was calculated for both time points. These derived metrics included the neutrophil-to-lymphocyte ratio (NLR), platelet-to-lymphocyte ratio (PLR), lymphocyte-to-monocyte ratio (LMR), systemic immune-inflammation index (SII; platelets × neutrophils/lymphocytes), prognostic nutritional index (PNI; 10 × serum albumin [g/dL] + 0.005 × total lymphocyte count), and the systemic inflammation response index (SIRI; neutrophils × monocytes/lymphocytes).

The primary outcome of the study was the occurrence of an anastomotic leak (AL), which was rigorously defined using a composite endpoint. A leak was considered present if any of the following criteria were met: clinical or radiological evidence of a communication between the intestinal lumen and the peritoneal cavity or exterior; the identification of a peri-anastomotic fluid collection on cross-sectional imaging suggestive of enteric origin; or the direct endoscopic or radiological visualization of an anastomotic defect before stoma reversal [4, 24].

### Statistical analysis and model development

Descriptive statistics were used to summarize study variables. Categorical variables are presented as absolute frequencies and percentages [*n* (%)]. The normality of continuous variables was assessed using the Kolmogorov–Smirnov test and visual inspection of Q-Q plots. Accordingly, continuous data are expressed as mean ± standard deviation [SD] for normally distributed variables or median (interquartile range [IQR]) for non-normally distributed data. Associations between categorical variables and the primary outcome were analyzed using the chi-square test. Measure of association was expressed as odds ratios (OR) with corresponding 95% confidence intervals (95% CI).

### Data preprocessing

The management of missing values followed a rigorous validation protocol to ensure the high fidelity of the machine learning inputs. We evaluated several imputation strategies, including Multiple Imputation by Chained Equations (MICE) using Predictive Mean Matching (PMM), Classification and Regression Trees (CART), and LASSO-based models. A comparative sensitivity analysis was predefined to determine the impact of these techniques on model metrics. The protocol established that, should a significant degradation in model calibration or predictive performance (AUC-PR) be observed due to the introduction of non-physiological noise in the time-sensitive inflammatory kinetics, a complete-case analysis would be prioritized. Consequently, following this internal validation, all models were trained and validated exclusively on records with complete laboratory and clinical data to maintain the biological integrity of the biomarkers.

### Data partitioning

The entire cohort was first divided into two temporally distinct groups (January 2012–December 2023 and January 2024–December 2025) for model development and subsequent temporal validation. Within the development cohort, the dataset was randomly partitioned into three subsets to ensure robust model training and evaluation: 70% of cases were allocated for training, 15% for hyperparameter tuning and internal validation, and the remaining 15% was reserved as an independent test set for final, unbiased performance assessment.

### Temporal external validation cohort

To evaluate temporal transportability and potential performance drift, the final selected and calibrated model was definitively locked in January 2024 without any further modification or retraining. All consecutive eligible patients treated after this date were prospectively evaluated using the locked model. This cohort was completely segregated from any phase of model development, tuning, calibration, or threshold selection and constitutes a TRIPOD Type 3 (temporal external) validation cohort.

### Class imbalance handling

The dataset exhibited significant class imbalance, with AL representing a minority class. To mitigate the associated risk of model bias toward the majority class and ensure clinically relevant sensitivity for leak detection, we implemented a dual-strategy approach. First, during model training, we applied cost-sensitive learning through instance weighting. The weight ratio was selected to approximate the inverse of the observed event prevalence in our cohort and to reflect the higher clinical cost of missing a true leak compared to a false alarm. Second, we prioritized the Area Under the Precision-Recall Curve (AUC-PR) over the Area Under the Receiver Operating Characteristic Curve (AUC-ROC) as our primary evaluation and optimization metric, as it provides a more realistic assessment of model performance in imbalanced scenarios by focusing on the correct identification of the rare, but critical, positive events.

### Machine learning algorithms and hyperparameter optimization

Five machine learning architectures, including Regularized Logistic Regression (RLB), Gradient Boosting (GBM), Random Forest (RF), and Deep Neural Networks (NN) Stacked Ensemble (Ensemble), were optimized using a random grid search with fivefold cross-validation. Hyperparameter tuning targeted optimal L1/L2 penalties, tree depth (3–30), and network topologies to maximize the Area Under the Precision-Recall curve (AUCPR). A Stacked Ensemble was then constructed using the best-performing models via logistic meta-learning. To prevent overfitting, all models utilized early stopping based on a convergence tolerance of 10 ^4^.

### Model validation and performance assessment

The predictive performance of all optimized models was rigorously evaluated on the held-out test set. Discrimination was assessed using AUC-ROC and AUCPR. Calibration was evaluated visually using calibration plots and quantitatively with the Brier Score and Expected Calibration Error (ECE).

### Statistical comparison and significance testing

Formal statistical comparisons between model performances were conducted using DeLong's test for paired AUC-ROC comparisons, with significance level set at *p* < 0.05. For multiple comparisons, we applied false discovery rate correction. The 95% confidence intervals for all performance metrics were calculated using 2000 bootstrap resamples.

### Model interpretation and feature analysis

Global model interpretability was assessed by analyzing variable importance scores. For non-linear models, SHapley Additive exPlanations (SHAP) summary plots visualized feature effects.

### Probability calibration and threshold optimization

To ensure the clinical reliability of the predictions across the 13-year study period, we performed a formal calibration and drift analysis. The final model was recalibrated using Platt Scaling, where a RLB model was fitted to the predicted log-odds. Calibration quality was quantified using the Brier Score and the calibration slope and intercept. Furthermore, a temporal drift analysis was conducted to verify the stability of the model’s performance and feature importance over time, ensuring that changes in perioperative care (e.g., evolving ERAS protocols) did not compromise the model's predictive integrity.

Thresholds were optimized using Youden’s index and F1-score.

### Clinical utility and decision curve analysis

The net clinical benefit was quantified across clinically plausible risk thresholds (0–20%) using Decision Curve Analysis (DCA), comparing model-based strategies against default interventions using the standardized net benefit formula.

### Software implementation

All analyses were performed using R software (version 4.3.3) with the h2o package for machine learning implementation. The pROC, PRROC, and rmda packages were utilized for ROC/PR analysis, calibration plots, and decision curve analysis, respectively.

## Results

### Patient cohort

**Fig. 1:**
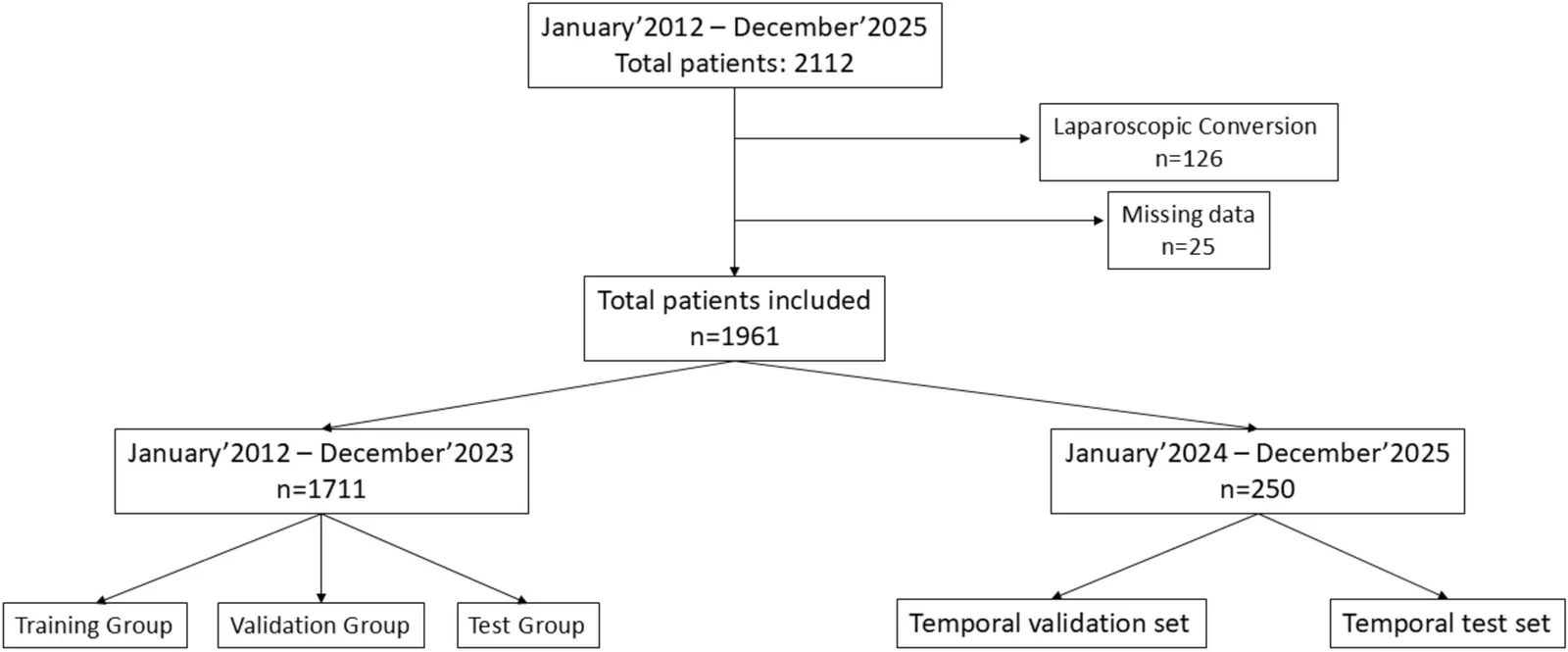
Flowchart of patient selection and study cohort allocation

**Fig. 2:**
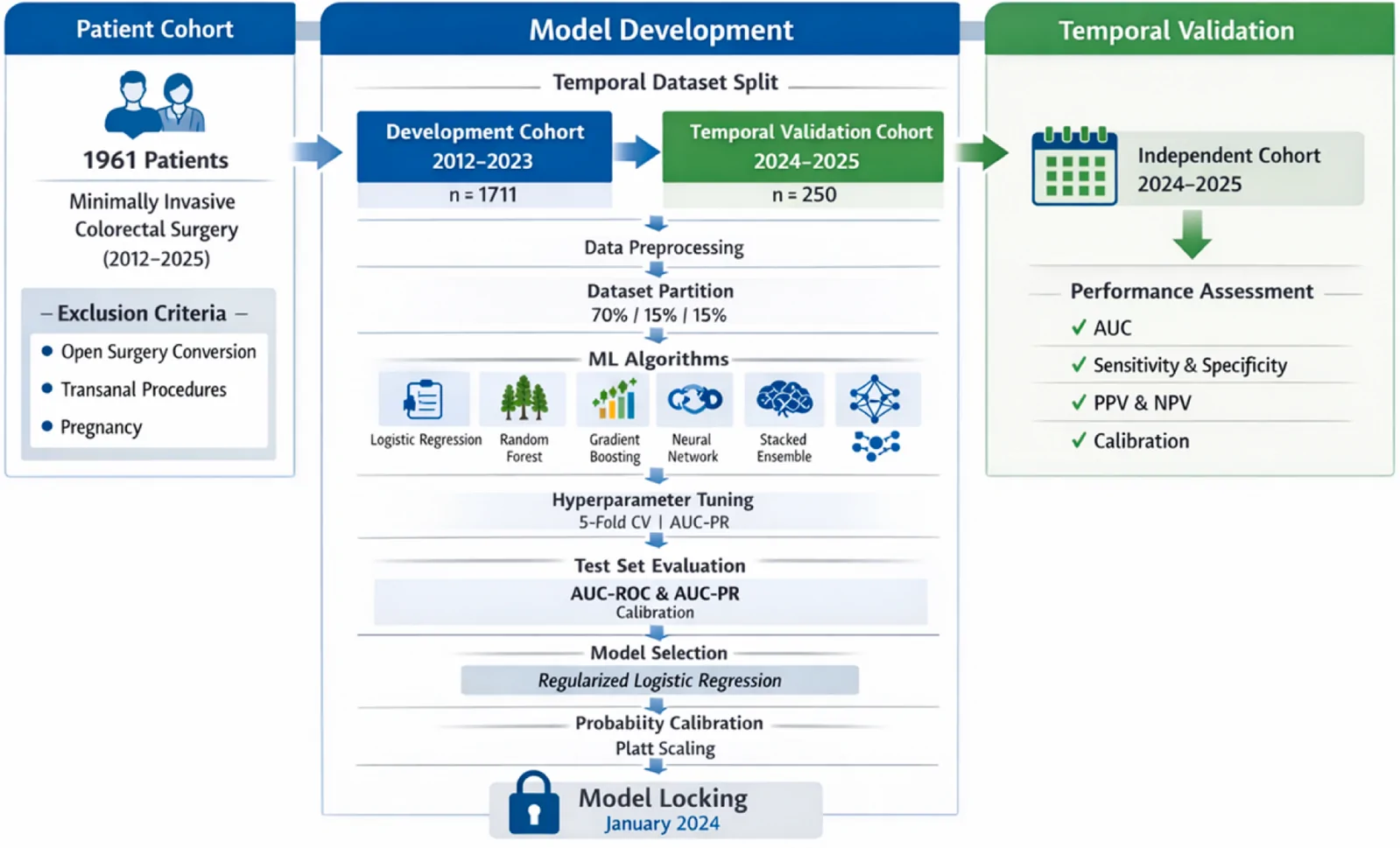
Study design and machine learning workflow. Flow diagram illustrating patient selection, dataset partitioning, and model development process

A total of 2,038 patients underwent elective minimally invasive colorectal cancer surgery during the study period and were screened for inclusion in the development cohort (2012–2023). Of these, 327 patients (16.0%) were excluded due to incomplete 24-h postoperative laboratory analytics or clinical records, resulting in a final complete-case development cohort of 1,711 consecutive patients (Fig. 1). For the prospective temporal validation cohort (2024–2025), 261 patients were initially screened, and 11 patients (4.2%) were excluded due to incomplete data, leaving a final validation cohort of 250 patients (Fig. 2, Table 1).

**Table 1:** Summary of patient characteristics, comorbidities, surgical technique, and perioperative laboratory values

	Normal *N* = 1544	Anastomotic leak *N* = 167	OR (95% CI)	*p*-value
Age (years)	72 (23–96)	69 (36–83)	–	0.014
Gender (female)	621 (40.2%)	40 (24%)	0.46 (0.32–0.68)	< 0.001
BMI (kg/m^2^)	27.05 (12.24–49.64)	26.45 (17.94–38.51)		0.572
Heart failure	47 (3%)	6 (3–6%)	1.19 (0.92–1.12)	0.639
COPD	193 (12.5%)	27 (16.2%)	1.35 (0.87 –2.09)	0.181
Chronic renal failure	83 (5.4%)	5 (3%)	0.54 (0.22 –1.36)	0.266
Diabetes	397 (25.7%)	40 (24%)	0.91 (0.63 –1.32)	0.709
Smoking	163 (10.6%)	24 (14.4%)	1.42 (0.9–2.26)	0.150
HTA	806 (52.2%)	82 (49.1%)	0.88 (0.64–1.22)	0.464
Peripheral artery disease	36 (2.3%)	10 (6%)	2.67 (1.3–5.48)	0.011
Cerebro-vascular disease	69 (4.5%)	8 (4.8%)	1.08 (0.51 –2.28)	0.844
Gastroduodenal ulcer	23 (1.5%)	9 (5.4%)	3.77 (1.71 –8.28)	0.003
Severe liver disease	20 (1.3%)	6 (3.6%)	2.84 (1.12 7.17)	0.035
Metastases	156 (10.1%)	22 (13.2%)	1.35 (0.84 –2.18)	0.229
Preoperative blood transfusion	94 (6.1%)	15 (9%)	1.52 (0.86 –2.69)	0.179
ASA				0.113
I	55 (3.2%)	9 (5.4%)		
II	761 (49.3%)	70 (41.9%)		
III	712 (46.1%)	84 (50.3%)		
IV	16 (1%)	4 (2.4%)		
Charlson’s Index	6 (2–13)	5 (2 – 9)		0.919
Neutrophils	4.37 (0.97–20.1)	4.69 (2.28 – 14.48)		0.001
Lymphocytes	1.63 (0.24–3.65)	1.87 (0.48–3.89)	–	0.491
Monocytes	0.59 (0.09–2.01)	0.69 (0.25–1.739	–	< 0.001
Platelets	238 (53–606)	266.5 (138–732)	–	0.077
NLR	4.96 (0.44–49.21)	9.69 (1.6 (45.71)	–	< 0.001
MLR	1.91 (0.01–12.46)	1.77 ((0.43–10)	–	< 0.001
PLR	172.73 (24.35–1307.69)	252.88 (78.18–2370)	–	< 0.001
SII	1068.48 (119.57–17,798.45)	2390.39 (412.817,398.87)	–	< 0.001
SIRI	1.57 (0.01–82.79)	2.05 (0.29–114.74)		< 0.001
Hb (g/dl)	12.8 (6.2–18.7)	12.35 (8.5–16)	–	0.446
Albumin	4 (1–9–5.7)	4.05 (2–5.1)	–	0.094
Preoperative blood transfusion	94 (6.1%)	15 (9%)	1.52 (0.86–2.69)	0.179
Mechanical & antibiotic intestinal preparation	213 (13.8%)	12 (7.2%)	0.48 (0.26–0.89)	0.015
Surgical approach				
Laparoscopy	1101 (71.3%)	95 (56.9%)	0.53 (0.38–0.74)	< 0.001
Robotic	7 (0.5)	0	–	1
Stoma	176 (11.4%)	23 (13.8%)	1.24 (0.78–1.98)	0.373
Operative time (min)	145 (33–450)	165 (55–450)		0.001
Class of anastomosis				
L-L	740 (49.9%)	69 (41.43%)		0.043
L–T	18 (1.2%)	5 (3%)		
T-L	10(0.6%)	3 (1.8%)		
T-T	776(50.3%)	90 (53.9%)		
Type of anastomosis				0.007
Ileo-colic	701 (45.4%)	61 (3.6%)		
Colo-colic	78 (5.1%)	16 (9.6%)		
Ileorectal	188 (91.8%)	17 (8.3%)		
Colorectal (10–15 cm)	362 (23.4%)	35 (21%)		
Colorectal (5–10 cm)	127 (8.2%)	24 (14.4%)		
Colorectal (< 5 cm)	88 (5.7%)	14 (8.4%)		
Postoperative Hb (g/dl)				
Neutrophils*	8.6 (0.31–26.32)	10.27 (2.59–26.84)		< 0.001
Lymphocytes*	0.88 (0.04–28)	0.79 (0.23–3)		0.159
Monocytes*	0.59 ((0.01–3.04)	0.63 (0.11–3.44)		0.037
Platelets	208 (47–782)	212.5 (103–596)		0.117
NLR*	10.02 (0.3–101.93)	12.6 (2.45–48.63)		< 0.001
MLR*	0.66 (0.01 (87.79)	2.05 (0.29–114.74)		0.001
PLR*	238.55 (11–2575)	272.61 (59.47–1221.74)		0.009
SII*	2034.91 (17–27,316)	2679 (618.31–		< 0.001
SIRI*	5.6 (0.03–89.63)	7.82 (0.95–32.22)		< 0.001
CRP*	67.9 (1.2–552.2)	84.25 (6.1–397.8)		< 0.001

* indicate the variable values in the first blood test taken 24 hours after the procedure

Following initial data preprocessing, it was observed that significant non-physiological noise was introduced into the time-sensitive inflammatory kinetics at 24 h by imputed values, leading to a demonstrable degradation of model performance. Consequently, to ensure the biological integrity of the biomarkers is preserved and high-fidelity inputs are maintained, it was decided that only complete cases would be utilized for the final model training and validation phases.

AL occurred in 167 patients (9.8%). Patients who developed AL were younger (median 69 vs. 72 years, *p* = 0.014), more frequently male (76.0 vs. 59.8%, *p* < 0.001), and had higher rates of peripheral artery disease (6.0 vs. 2.3%, *p* = 0.011), gastroduodenal ulcer (5.4 vs. 1.5%, *p* = 0.003), and severe liver disease (3.6 vs. 1.3%, *p* = 0.035). Longer operative times (median 165 vs. 145 min, *p* = 0.001), lower laparoscopic completion rates (56.9 vs. 71.3%, *p* < 0.001), and lower utilization of combined mechanical and antibiotic bowel preparation (7.2 vs. 13.8%, *p* = 0.015) were also observed in the leakage group.

Regarding rectal resections (*n* = 353), the analysis accounted for the impact of diverting stomas on AL presentation. The total incidence of AL in the stoma subgroup was 12.5%. This included 14 cases (7.6%) of Grade A (asymptomatic) leakage and 9 cases (4.9%) of Grade C leakage. The detection of these events—particularly the Grade C cases occurring despite fecal diversion—highlights the model's capacity to identify high-risk inflammatory profiles that precede or transcend overt clinical peritonitis.

Temporal analysis revealed significant shifts in clinical practice and outcomes. The second period was characterized by the widespread adoption of combined mechanical bowel preparation and oral antibiotics (*p* < 0.001), which coincided with a significant reduction in the anastomotic leakage rate (*p* < 0.001). In the second period, the odds of developing an anastomotic leak were significantly lower (OR 0.26; 95% CI 0.12–0.56).

### Model development and internal validation

**Fig. 3:**
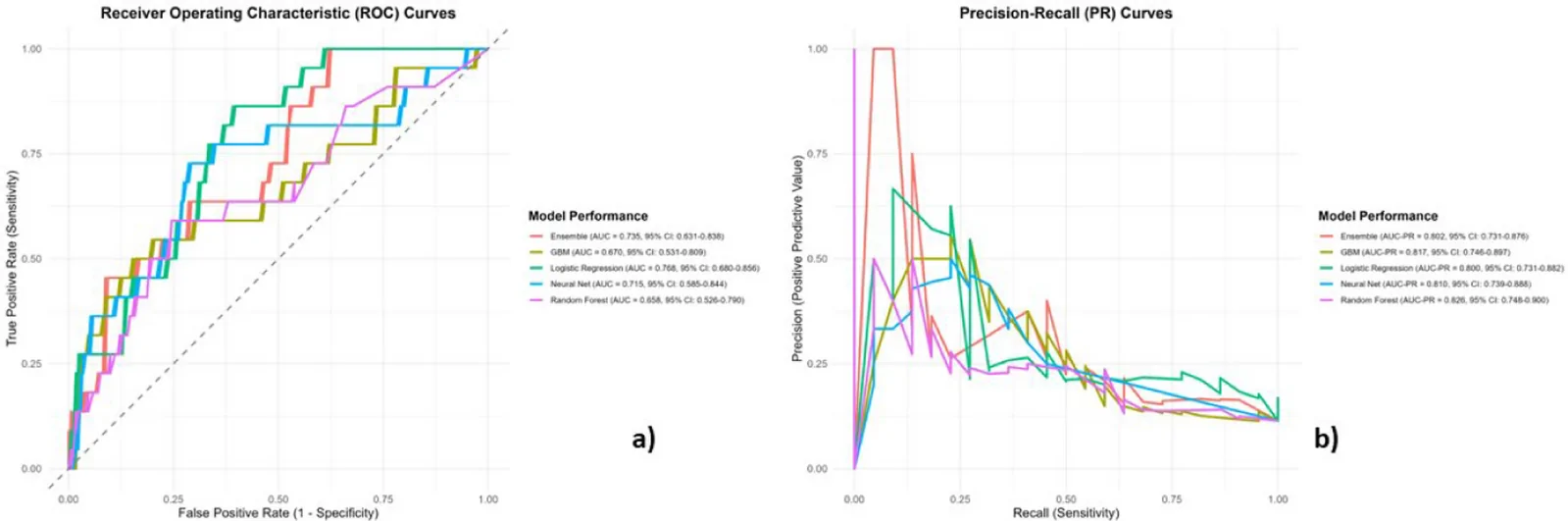
Discriminative performance of machine learning models in the internal test set **a** Receiver operating characteristic (ROC) and **b** precision–recall curves comparing candidate models

Five machine learning models were developed using a 70%/15%/15% split with class weighting (10:1) to address class imbalance. The RLB model achieved the highest discrimination on the held-out test set (*n* = 255), with an AUC-PR of 0.859 (95% CI, 0.821–0.897) and an AUC-ROC of 0.819 (95% CI, 0.781–0.857). Random Forest (RF) and Stacked Ensemble (Ensemble) models showed similar performance (AUC-PR 0.834 and 0.831, respectively), while the Neural Network (NN) exhibited significantly lower outcomes (AUC-PR 0.729) (Fig. 3a). Pairwise DeLong tests confirmed a superior AUC-ROC for RLB versus DNN (*p* = 0.019); no other differences remained significant.

Subgroup analysis confirmed consistent discrimination, with an AUC-ROC of 0.891 for colon and 0.794 for rectal resections. Although the AUCPR in the rectal group (0.292) was lower than in the colon group (0.822), this drop mathematically reflects the lower baseline prevalence of events in this specific subcohort rather than a true loss of discriminative ability, as precision-recall metrics are highly sensitive to class imbalance. The clinical utility remained uncompromised, with the NPV remaining consistently high above 95% across both anatomical locations, supporting the model's generalizability.

Several machine learning architectures demonstrated comparable discrimination. However, the RLB achieved the highest AUC-PR while maintaining greater interpretability and simplicity (Fig. 3b). Thus, the RLB was chosen as the final model for probability calibration and subsequent temporal validation.

### Calibration and threshold selection

Calibration performance differed across models depending on the calibration method applied. Platt scaling provided better calibration for simpler models such as logistic regression and random forest, whereas isotonic regression yielded lower Brier scores and ECE values for more flexible models including gradient boosting, neural networks, and the ensemble model.

Overall Brier scores ranged from 0.0898 to 0.0975 across models, with ECE values between 0.0357 and 0.0582, indicating acceptable calibration performance.

After calibration, the discrimination of the RLB achieved the highest AUC-ROC (0.768), followed by the ensemble model (0.756) and NN (0.706). Precision-recall analysis showed similar performance across models, with AUC-PR values ranging from 0.795 to 0.826.

**Fig. 4:**
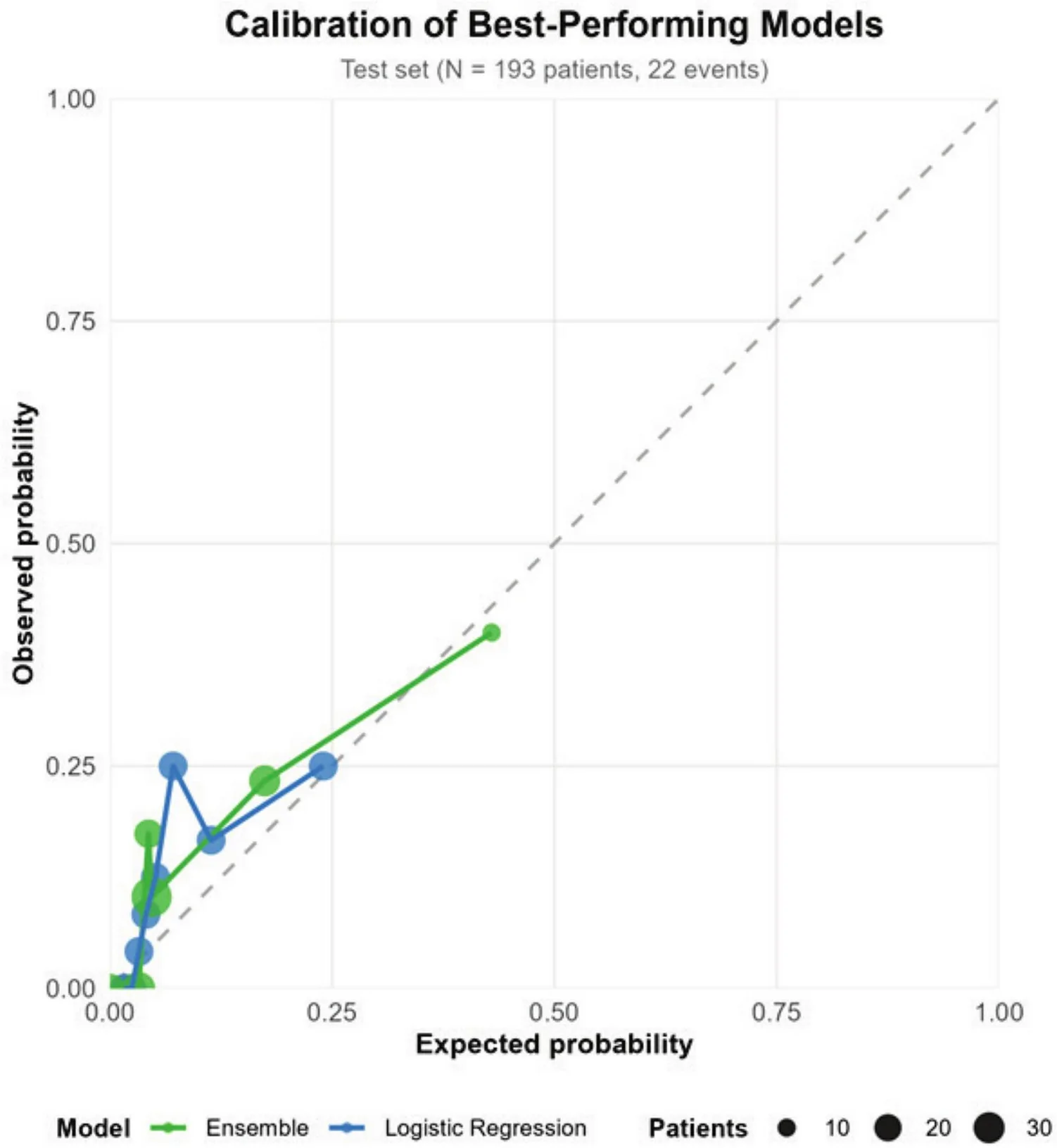
Calibration plots for the best-performing machine learning models in the test set. The graph displays the relationship between expected (predicted) probabilities and observed probabilities of the outcome for the Ensemble model (green line) and Logistic Regression model (blue line). The dashed diagonal line represents perfect calibration, where predicted probabilities exactly match observed outcomes. Point sizes are proportional to the number of patients in each risk group (10, 20, and 30 patients). The test set included 193 patients with 22 events. Models demonstrating good calibration show points closely aligned with the 45-degree reference line, indicating accurate probability predictions across the range of risk estimates (color figure online)

Using the Youden index, RLB achieved the highest sensitivity (0.864) while maintaining moderate specificity (0.608), suggesting improved ability to identify high-risk patients compared with other models (Fig. 4).

The calibration intercept was 0.51 (95% CI 0.23–0.78) and the calibration slope was 0.99 (95% CI 0.86–1.13), indicating good agreement between predicted and observed probabilities.

### Model interpretation and clinical utility

**Fig. 5:**
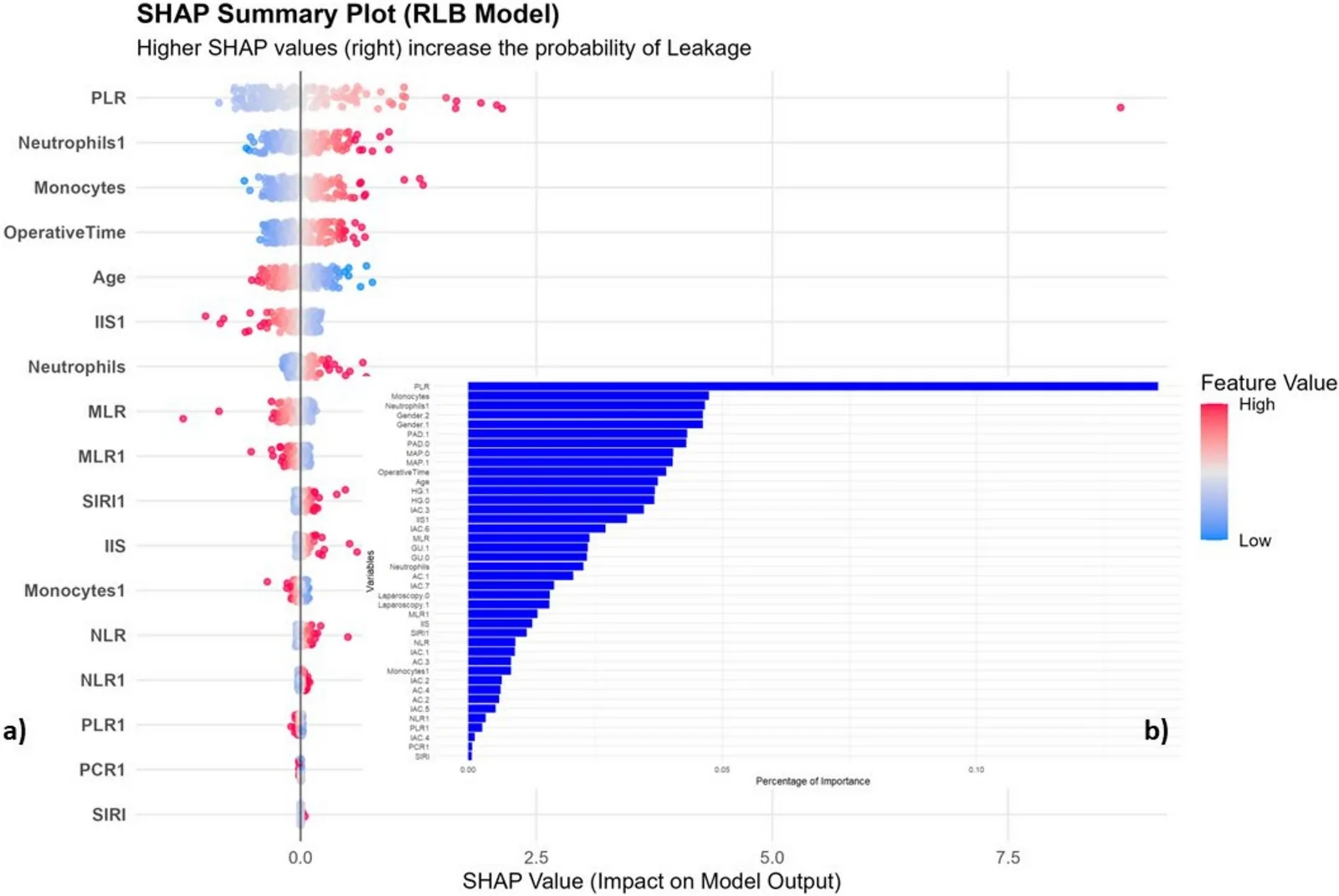
SHAP (SHapley Additive exPlanations) summary plot illustrating feature importance and directional impact on the predicted probability of leakage for the Regularized Logistic Regression (RLB) model.** a** Beeswarm plot displaying the distribution of SHAP values for each feature, where each dot represents a patient. Features are ranked vertically by importance (top to bottom). **b** Bar chart showing the mean absolute SHAP values for each feature, representing overall feature importance.

**Fig. 6:**
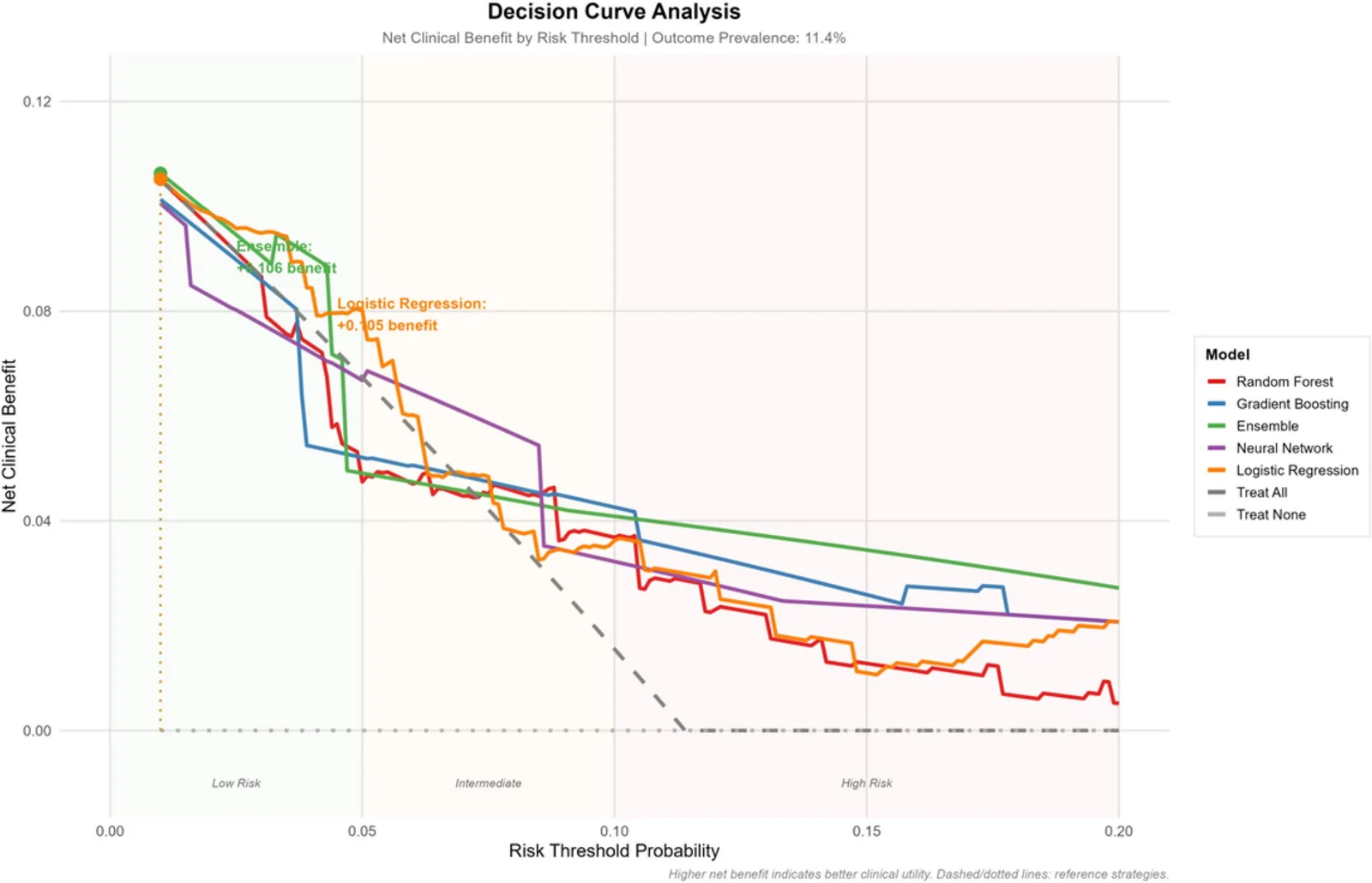
Decision curve analysis (DCA) comparing the clinical utility of machine learning models across different risk threshold probabilities

The final Regularized Logistic Regression (RLB) model was optimized using a Ridge penalty (lambda = 0.0287), achieving an AUC-ROC of 0.819 and an AUCPR of 0.859. Feature importance and directional impact were evaluated using SHAP (SHapley Additive exPlanations) values (Fig. 5a). The analysis identified the Platelet-to-Lymphocyte Ratio (PLR) as the most influential predictor, followed by Monocyte count and Gender (Fig. 5b) 0. Specifically, the SHAP summary plot (Fig. 5a) revealed that higher values of PLR and absolute Monocyte counts at 24 h were associated with an increased probability of anastomotic leakage. Other systemic markers, including the Systemic Inflammation Response Index (SIRI) and CRP, also contributed to the model’s predictive weight. Among clinical variables, longer operative times and an open surgical approach (versus laparoscopy) were identified as significant factors driving higher risk estimates. This combination of biological and surgical features provided a calibrated and stable prediction, as confirmed by the consistency between training (AUC 0.806) and cross-validation metrics (AUC 0.734). Decision curve analysis (DCA) demonstrated a net clinical benefit across risk thresholds between 5 and 20%, supporting the model's potential clinical applicability (Fig. 6).

### Temporal validation

To assess the model's stability over time, a drift analysis was performed between the derivation (2012–2023) and validation (2024–2025) cohorts (Table 2). Despite a decrease in the AL incidence rate in the temporal validation group (2.8 vs. 9.8%), the model maintained high calibration and discriminative power. No significant feature drift was observed in the primary predictors, confirming that the biological signal captured by the model remains a consistent indicator of AL across different clinical periods.

**Table 2:** Summary of predictor variables utilized in the model for temporal validation

	Normal *N* = 243	Anastomotic Leak *N* = 7	OR (95% CI)	*p*-value
Age (years)	73 (18–91)	66 (56–85)	–	0.742
Gender (female)	91 (34.4%)	–	–	**0.050**
Risk factors				
Peripheral artery disease	9 (3.7%)	1 (14.3%)	4.33 (0.47–39.87)	0.251
Gastroduodenal ulcer	2 (0.8%)	–	–	1
Severe liver disease	5 (2.1%)	–	–	1
Preoperative laboratory values				
Neutrophils (× 10⁹/L)	4.12 (1.28–19.03)	4.67 (2.25–8.53)	–	0.652
Lymphocytes (× 10⁹/L)	1.73 (0.5–4.21)	1.57 (0.71–10.26)	–	0.633
Monocytes (× 10⁹/L)	0.62 (0.17–1.48)	0.42 (0.32–0.51)	–	**0.001**
Platelets (× 10⁹/L)	235 (63–544)	229 (90–297)	–	0.240
NLR	2.49 (0.80–27.99)	2.42 (0.22–12.01)	–	0.905
MLR	0.34 (0.14–1.88)	0.27 (0.05—0.51)	–	0.064
PLR	130.41 (38.48–622.03)	101.27 (22.32–328.17)	–	0.473
SII	540.30 (120.28–4645.56)	430.52 (51.34–2799.28)	–	0.434
SIRI	1.45 (0.36–35.82)	0.94 (0.11–4.33)	-	0.118
Mechanical & antibiotic intestinal preparation	–	7 (2.8%)	0.99 (0.98–1.00)	1
Laparoscopy	228 (93.8%)	6 (85.7%)		
Operative time (min)	163 (54 – 385)	165 (78 – 210)	-	0.594
Class of anastomosis				0.834
L-L	110 (45.3%)	2 (28.6%)		
L–T	1 (0.4%)	–		
T-L	1 (0.4%)	–		
T-T	131 (53.9%)	5 (71.4%)		
Postoperative laboratory values*				
Neutrophils (× 10⁹/L)	9.26 (2.13–21.56)	8.45 (5.05–11.09)	–	0.476
Lymphocytes (× 10⁹/L)	0.80 (0.19–3.35)	1.03 (0.74–10.38)		**0.037**
Monocytes (× 10⁹/L)	0.6 (0.07–2.27)	0.85 (0.34–1.48)		**0.042**
Platelets (× 10⁹/L)	209 (62–624)	225 (135–339)		0.760
NLR	12.14 (1.90–56.48)	7.74 (0.99–14.99)	–	**0.021**
MLR	0.75 (0.14–3)	0.79 (0.14–1.15)	–	0.799
PLR	270.25 (35.03–1223.53)	179.05 (21.68–314.85)	–	0.090
SII	2553.86 (366.75–26,379.29)	1455.66 (223.05––3491.85)		0.058
SIRI	6.80 (0.87–42.36)	6.65 (1.47–9.37)	–	0.428
PCR (mg/L)	40.20 (3.80–292.8)	30.2 (5.5–13.8)	–	0.412

* indicate the variable values in the first blood test taken 24 hours after the procedure

Bold indicates statistical significance

Continuous variables are expressed as median (range) and categorical variables as n (%). Neutrophils, lymphocytes, monocytes and platelets are expressed as × 10⁹/L.

*BMI* body mass index, *COPD* chronic obstructive pulmonary disease, *HTA* hypertension, *ASA* American Society of Anesthesiologists physical status classification, *Hb* hemoglobin, *CRP* C-reactive protein, *NLR* neutrophil-to-lymphocyte ratio, *MLR* monocyte-to-lymphocyte ratio, *PLR* platelet-to-lymphocyte ratio, *SII* systemic immune-inflammation index, *SIRI* systemic inflammation response index, *OR* odds ratio, *CI* confidence interval

The final calibrated RLB was locked with a pre-specified Youden-derived threshold of 0.1258. This model was prospectively applied without modification to a temporal validation cohort of 250 consecutive patients operated on between February 2024 and December 2025.

During this validation period, a significant shift in local epidemiology was observed: the incidence of AL decreased to 2.8% (7/250), representing a relative reduction of over 70% compared to the development cohort. Under this lower prevalence scenario, the model maintained acceptable discrimination with an AUC of 0.73 (95% CI 0.54–0.92). Using the pre-specified threshold, clinical performance yielded a sensitivity of 57.1% and a specificity of 60.1%. Notably, despite the change in event rate, the negative predictive value (NPV) remained robust at 97.9% (95% CI 95.1%–99.1%), confirming the model's reliability in identifying low-risk patients. However, the lower contemporary incidence resulted in a substantial increase in false-positive classifications (*n* = 97), leading to a positive predictive value of 4.0%.

Reliability analysis confirmed the technical robustness of the RLB model. The calibration metrics showed a slope of 0.987, indicating a near-optimal relationship between predicted and observed risks. A calibration intercept of 0.5054 was observed, suggesting a slight initial tendency toward underestimating absolute risk, which was addressed during the final probability mapping. The overall predictive accuracy was further supported by a Brier Score of 0.094, reinforcing the model’s stability for clinical risk assessment. Importantly, post hoc analysis confirmed that the original threshold of 0.1258 remained statistically optimal according to the Youden index within the contemporary cohort. While higher thresholds would have markedly reduced false-positive alerts, the prospectively defined criteria were maintained to preserve the integrity of the study design.

## Discussion

This study developed and temporally validated a machine learning model integrating preoperative variables and early postoperative inflammatory biomarkers to predict AL following minimally invasive colorectal surgery. The calibrated regularized RLB model demonstrated good discrimination (AUC 0.819 in the development cohort and 0.73 in temporal validation) and, most importantly, a high negative predictive value (97.9%). These findings suggest that routinely available clinical and laboratory data within the first 24 h after surgery can support early postoperative risk stratification. Despite a 70% reduction in leak incidence during the validation period, model discrimination and calibration remained stable.

A key aspect of the model is the incorporation of early systemic inflammatory markers, including NLR, SIRI, monocyte count, and 24-h CRP. These biomarkers reflect the host inflammatory response to surgical stress and may capture early deviations from normal postoperative recovery [19, 25–34]. Experimental and clinical studies suggest that anastomotic failure may begin within the first 48 h after surgery, when local ischemia, impaired perfusion, and early inflammatory responses disrupt anastomotic healing [4, 18, 19, 24]. Previous investigations have also reported that early physiological changes, such as reductions in peri-anastomotic intramucosal pH or elevations in C-reactive protein levels, may be detectable as early as the first postoperative day [17, 35]. In line with these observations, postoperative inflammatory markers consistently ranked among the strongest predictors in our model, suggesting that early systemic inflammatory dynamics may provide clinically relevant signals preceding overt clinical deterioration.

The threshold for classification was determined using Youden’s Index to provide a statistically balanced baseline. While clinical applications focused on early discharge must prioritize the minimization of false negatives, our model achieved a Negative Predictive Value (NPV) of 97.9% at the optimized threshold. This indicates that the systemic inflammatory signal captured at 24 h provides a sufficiently high safety margin to identify patients at low risk for anastomotic leakage, fulfilling the requirements for a clinical 'rule-out' tool without the need for arbitrary threshold shifts that would disproportionately increase the false-positive rate.

Beyond statistical discrimination, the clinical value of a predictive model lies in its ability to influence postoperative decision-making. Decision curve analysis demonstrated a consistent net clinical benefit across risk thresholds between 5 and 20%. Decision curve analysis has been proposed as a method to evaluate the clinical utility of prediction models by incorporating the relative consequences of false-positive and false-negative decisions, thereby translating statistical performance into potential clinical benefit [36, 37]. In practical terms, the high negative predictive value observed in the validation cohort suggests that the model may function as an early postoperative rule-out tool. This stable performance across anatomical sites is further supported by our subgroup analysis; despite the mathematical reduction in AUCPR in rectal cases due to lower event prevalence, the high negative predictive value was maintained, reinforcing the model's safety profile regardless of the surgical location. Patients classified as low risk within the first 24 h could be considered for accelerated discharge pathways within enhanced recovery programs, whereas higher risk patients may benefit from closer monitoring or early diagnostic imaging.

These findings may also have implications for emerging ambulatory or short-stay colorectal surgery pathways. In this context, early biomarker-based risk stratification may provide an additional safety layer to support early discharge decisions.

An important strength of this study is the temporal validation performed in a subsequent cohort of patients treated after model development. During this period, the incidence of AL decreased markedly from 9.8% to 2.8%, illustrating how evolving surgical practices may introduce dataset shift in predictive models. The marked decline in anastomotic leak rates in the more recent period may be largely attributed to the routine adoption of oral bowel preparation and antibiotic prophylaxis. Our observation reinforces existing evidence supporting the protective role of combined bowel preparation [2, 38–40], suggesting that its systematic application is a critical factor in optimizing postoperative outcomes and reducing the incidence of leakage. Although the lower event rate reduced the positive predictive value and increased the number of false-positive alerts, overall model discrimination remained acceptable and calibration remained stable. These findings highlight the importance of temporal validation in surgical prediction modeling and underscore the need to monitor model performance over time as clinical practice evolves [41–43].

Several limitations should be acknowledged. First, while this was a single-center study, its longitudinal design (2012–2025) introduces potential biases related to evolving surgical strategies, such as the refinement of ERAS protocols and the standardization of mechanical bowel preparation with oral antibiotics. However, this long-term perspective ensured a consistent high-volume environment where the model underwent strict temporal validation in a recent independent cohort. The stability of the model's performance despite these historical changes represents a robust test of its transportability. Second, the substantial reduction in AL incidence during the validation period (2.8%) resulted in a low positive predictive value and a relatively high number of false-positive alerts. Consequently, the model functions primarily as a rule-out tool, prioritizing a high negative predictive value (97.9%) to ensure patient safety in early discharge protocols. Third, although inflammatory markers like CRP and SIRI capture early host responses, they are non-specific indicators and were selected by our protocol only after sensitivity analyses showed that imputation of missing values introduced unacceptable non-physiological noise. Finally, this study serves as the pilot phase for an ongoing multicenter project, which will provide the necessary external validation to confirm the robustness of these findings across diverse clinical settings (Table 3).

**Table 3:** Comparative performance metrics of machine learning models for 24 h anastomotic leakage

Model	AUC-ROC	AUC-PR	Brier Score	ECE	Th	Sensitivity	Specificity	Precision	F1_Score
RF	0.66	0.83	0.098	0.058	0.088	0.591	0.754	0.236	0.338
GBM	0.68	0.81	0.092	0.036	0.096	0.545	0.801	0.261	0.353
NN	0.71	0.81	0.092	0.036	0.068	0.773	0.591	0.195	0.312
RGL	0.77	0.80	0.094	0.054	0.050	0.864	0.608	0.221	0.352
Ensemble	0.76	0.80	0.090	0.052	0.038	1.000	0.363	0.168	0.288

The optimal classification threshold was determined using Youden’s J statistic. Sensitivity, specificity, precision, and F1-score are reported at this optimal threshold

*AUC* area under the curve, *ROC* receiver operating characteristic, *PR* precision-recall, *ECE* expected calibration error, *Th* threshold, *RF* random forest, *NN* neural net, *GBM* gradient boosting machine, *RGL* regularized lineal model, *Ensemble*, stacket ensemble

In conclusion, this study developed and temporally validated a machine learning model integrating preoperative variables and early postoperative inflammatory biomarkers to predict anastomotic leakage after minimally invasive colorectal surgery. The model demonstrated good discrimination, calibration, and a high negative predictive value, supporting its potential use for early postoperative risk stratification. Such an approach may help guide postoperative monitoring and decision-making in patients undergoing colorectal resection.

## Data Availability

Anonymized data are available upon reasonable request to the corresponding author. Access to raw clinical data may be restricted to protect patient privacy and in accordance with institutional data sharing policies.
